# Increasing Case Numbers and Earlier Age at Diagnosis of Alveolar Echinococcosis: Insights from a 13-Year Retrospective Swiss Study

**DOI:** 10.3390/pathogens15050495

**Published:** 2026-05-04

**Authors:** Lasse Weikert, Sophie Kasmi, Michael Schneider, Mohamed Faouzi, Sabine Schmidt Kobbe, Christine Sempoux, Matthias Cavassini, Emeline Gauthiez, Emilie Uldry, Montserrat Fraga

**Affiliations:** 1 Service of Gastroenterology and Hepatology, Lausanne University Hospital, 1011 Lausanne, Switzerland; 2 Service of Visceral Surgery, Lausanne University Hospital, 1011 Lausanne, Switzerland; 3 Division of Biostatistics, Center for Primary Care and Public Health (Unisanté), 1011 Lausanne, Switzerland; 4 Department of Diagnostic Radiology and Interventional Radiology, Lausanne University Hospital, 1011 Lausanne, Switzerland; 5 Institute of Pathology, Lausanne University Hospital, 1011 Lausanne, Switzerland; 6 Service of Infectious diseases, Lausanne University Hospital, 1011 Lausanne, Switzerland

**Keywords:** alveolar echinococcosis, *Echinococcus multilocularis*, epidemiology, immunosuppression, Switzerland

## Abstract

Introduction: Alveolar echinococcosis (AE) is a rare zoonotic infection caused by *Echinococcus multilocularis*. In recent years, the number of reported cases has increased across Europe, including Switzerland. Methods: We conducted a retrospective observational study at Lausanne University Hospital (CHUV), including all AE cases diagnosed between 2010 and 2023 within the Departments of Gastroenterology and Hepatology, Visceral Surgery, and Infectious Diseases. Results: Eighty patients with AE were identified. Half of them were male. The mean annual incidence was 0.53 cases per 100,000 population (range, 0.14–0.85), with a statistically significant increase over time in the canton of Vaud (*p* = 0.037). The median age at diagnosis was 61.5 years, showing a significant downward trend during the study period. At diagnosis, 45 patients (56.3%) were asymptomatic, and 17 (21.3%) were immunosuppressed. Compared with immunocompetent patients, immunosuppressed individuals more often presented asymptomatically, had significantly smaller hepatic lesions, and were more frequently seronegative. Conclusions: This study demonstrates evolving epidemiological patterns of AE in Switzerland, characterized by increasing incidence and a high proportion of asymptomatic cases. Immunosuppressed patients tended to present with milder disease. Our findings highlight the need for strengthened surveillance and support the ongoing efforts to establish a national AE registry.

## 1. Introduction

Alveolar echinococcosis (AE) is caused by the larval stage of the tapeworm Echinococcus multilocularis (EM). It primarily affects wild carnivores, such as foxes. Humans become infected by ingesting food contaminated with EM eggs, acting as accidental hosts and disrupting the parasite’s lifecycle [1].

AE is a neglected zoonotic disease recognized by the World Health organization (WHO), presenting significant challenges in both human and veterinary health [2]. As of 2010, it caused about 7771 deaths and 688,000 disability-adjusted life years (DALYs) worldwide [3]. Improved diagnostics and surveillance have increased case detection, making AE a growing public health concern [2].

For many decades, EM infection in foxes and human AE were largely restricted to Central Europe. In recent decades, extensive research has revealed a significant expansion of the disease into Northern, Eastern, and Western Europe [1,4]. This has been accompanied by a steady rise in its incidence, both globally and in Switzerland [5,6,7].

These epidemiological trends can be attributed to a variety of factors. First, EM is increasingly diagnosed due to improved awareness, enhanced surveillance systems and advanced diagnostic methods. Indeed, advances in imaging techniques, alongside refined serological tests, have enabled earlier detection, even in asymptomatic individuals [8]. Moreover, across Europe, the increase in human AE cases is largely attributed to expanding red fox populations, rising EM infection rates among animals, and the progressive colonization of urban areas by foxes [9,10,11,12]. In Switzerland, fox populations have increased nearly fourfold since the 1980s, with parasite prevalence reaching up to 65% in some regions [13,14].

AE typically remains asymptomatic for years and is often discovered incidentally on imaging. Symptoms appear late and vary with disease extension. Primarily affecting the liver, AE mimics malignancy, namely cholangiocarcinoma. In advanced stages, it infiltrates liver tissue, causing biliary or vascular obstruction. AE can also spread to distant organs like the lungs or the brain, complicating diagnosis and treatment [15].

Immunosuppressed individuals, tend to exhibit more aggressive and atypical forms of AE [16]. In these populations, the disease may progress rapidly with less typical imaging features, earlier extrahepatic dissemination, reflecting impaired immune containment of the parasitic proliferation [16,17].

**Figure 1:**
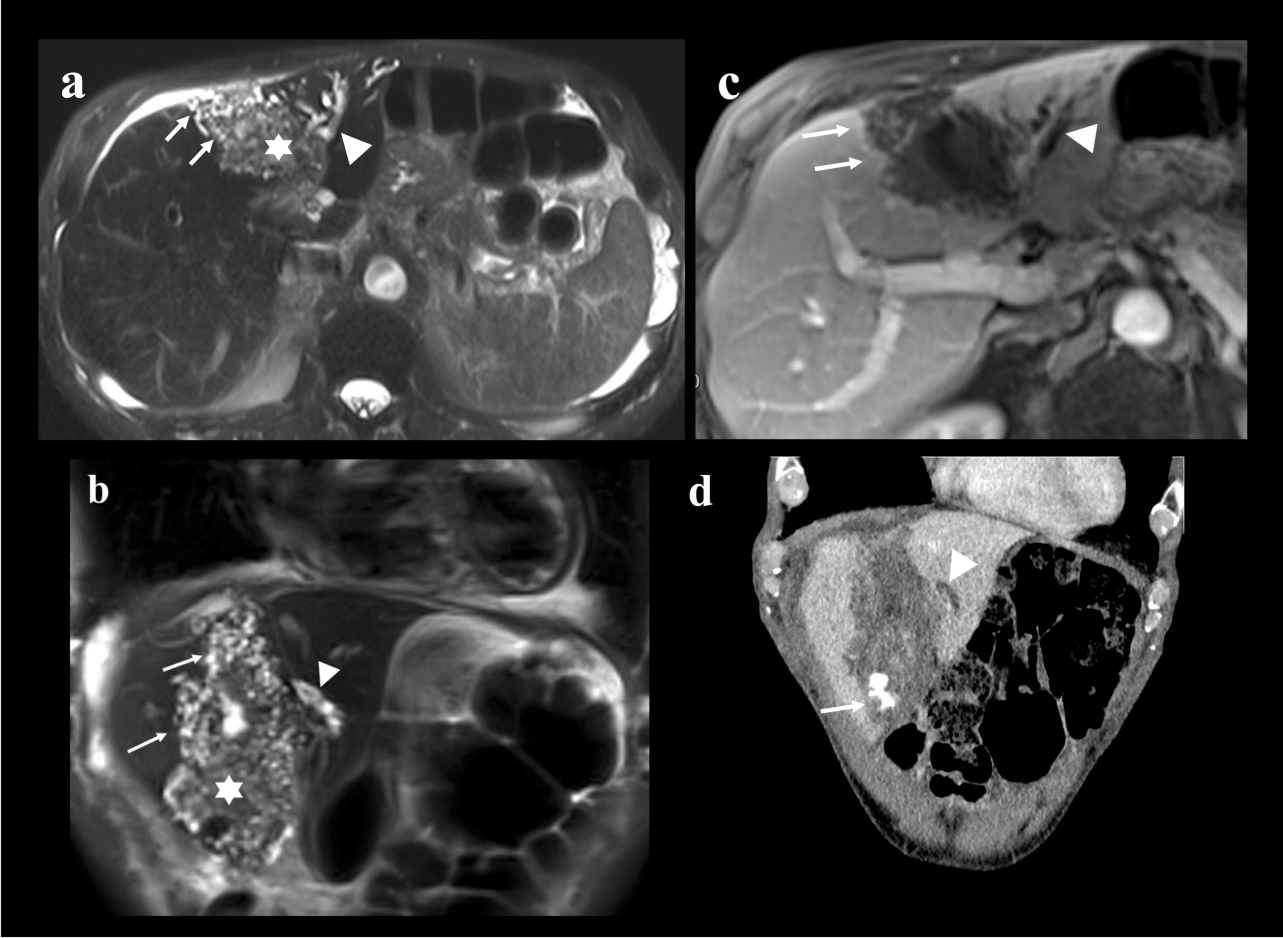
T2-weighted axial (**a**) and coronal (**b**) MR images show the characteristic multivesicular structure of alveolar echinococcosis (AE) located in the fourth liver segment detected in a 68-year-old man. The lesion is composed of multiple small cysts surrounding a solid component (**a**,**b**, stars) and a large irregular cyst. Note the extension into the left liver lobe with bile duct infiltration and consecutive biliary dilatation (**a**–**d**, triangles). The correspondent T1-weighted axial MR image (**c**) after intravenous gadolinium injection shows the typical faint enhancement at the periphery of the lesion (**c**, arrows) and the infiltrative character because of ill-defined margins, particularly well seen in the posterior part of this lesion, explaining the frequent misdiagnosis as a tumor. The coronal CT-image of the same patient (**d**) nicely demonstrates the typical coarse calcification of AE (**c**, arrow).

Diagnosis relies on a combination of imaging and serology. Ultrasonography (US) is the first-line examination, while magnetic resonance imaging (MRI) and computed tomography (CT) further characterize the lesion and its invasion of surrounding structures. The characteristic radiological features on CT and MRI are illustrated in Figure 1. Additional imaging (thoracic CT, brain MRI) is recommended to assess distant dissemination, while (Positron Emission Tomography-Computed Tomography) PET-CT is useful for determining the metabolic activity of lesions, which correlates with parasite viability [18].

**Figure 2:**
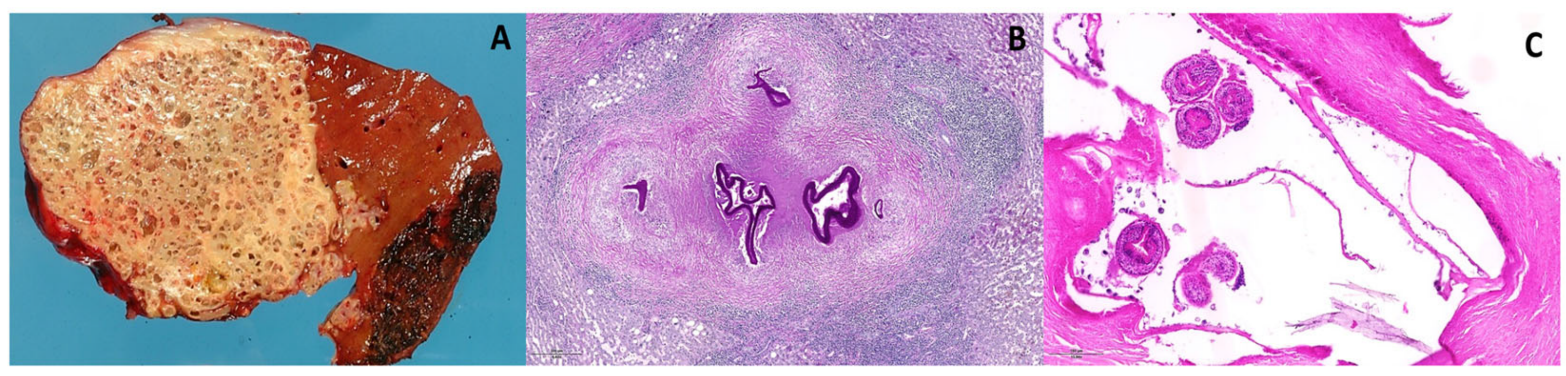
(**A**) Macroscopic picture with the characteristic microcystic aspect of the mass within the liver. (**B**) Microscopic features showing the florid confluent granulomatous reaction with a necrotic center containing PAS positive laminated membranes and a rim of lymphocytic inflammation (Periodic acid- Schiff stain, scale bar 300 mm). (**C**) Higher power focusing on the protoscoleces of Echinococcus multilocularis (Hematoxylin-eosin stain, scale bar 150 mm).

Serology, including ELISA and immunodot tests, complements imaging by confirming exposure and monitoring disease progression and activity [18]. Histopathological examination remains the gold standard for definitive diagnosis [19], revealing the characteristic multivesicular structure as seen in the macroscopic and microscopic views in Figure 2.

Over the past decades advances in treatment and early diagnosis have led to substantial improvements in AE survival rates [20]. The therapeutic approach to AE depends largely on the stage of the disease at diagnosis [19]. In early or localized stages, radical surgical resection of the hepatic lesion with R0 margins remains the treatment of choice and offers the best chance for cure [18,21]. Surgery is typically followed by prolonged adjuvant therapy with benzimidazoles (usually albendazole) to prevent recurrence. In cases where radical surgery is not feasible, long-term or lifelong antiparasitic therapy with benzimidazoles becomes the mainstay of treatment. These agents are parasitostatic rather than parasiticidal, requiring continuous administration to control disease progression [18,22]. In advanced or disseminated AE, palliative interventions such as biliary drainage and percutaneous procedures may be considered [23]. In selected cases, liver transplantation may be performed as a last resort; however, it is rarely undertaken due to the high risk of recurrence [24]. There is currently almost no more place for palliative surgery in AE treatment [18,19].

In Switzerland, where AE is an endemic disease. It’s incidence has risen significantly over the past decades, reportedly doubling between 1993–2000 and 2000–2005, going from 0.10/100,000 to 0.26/100,000, with an even sharper increase in subsequent years [13].

The actual numbers are likely significantly higher, as suggested by more recent data from the Swiss Echinococcosis Network estimating annual incidence to range from 0.58 to 1.33 per 100,000 population [25].

In this context, we aimed to assess the epidemiological and demographic trends of AE, as well as its clinical presentation, in a tertiary university hospital in Switzerland.

## 2. Methodology

### 2.1. Study Design and Setting

We conducted a retrospective non-interventional study, at Lausanne University Hospital (CHUV), covering the period from 1 January 2010 to 31 December 2023. The study included all patients who received care at CHUV and were managed for AE within at least one of the three primary departments involved in AE treatment: Gastroenterology and Hepatology, Visceral Surgery, or Infectious Diseases.

The study protocol was reviewed and approved by the local ethics committee (CER-VD, protocol number 2023-02283). All patients provided consent for the coded reuse of their data.

### 2.2. Patient Identification

Patient identification was carried out with the support of the hospital’s Data Science Unit, utilizing a multiparametric clinical data warehouse search engine. All cases of echinococcosis diagnosed during the study period, including cystic echinococcosis (CE) were collected to ensure comprehensive case identification. This approach was designed to ensure accurate classification of echinococcosis cases and to minimize the risk of misclassification or omission of AE cases.

Inclusion criteria: (1) Patients who had provided general consent (GC) for the reuse of their medical data, or, in the absence of GC, had signed a dedicated study-specific information sheet. (2) A confirmed diagnosis of AE following medical chart review by two investigators (L.W. and M.F.). Two diagnostic pathways were considered acceptable for confirming AE. The first was based on histopathological confirmation, demonstrating the presence of the parasite in a surgical specimen or biopsy, corresponding to the WHO definition of a “confirmed case”. The second was the combination of positive AE serology with characteristic imaging findings, meeting the criteria for a “probable case” according to WHO guidelines [19].

Exclusion criteria: (1) Uncertain or unconfirmed diagnosis of AE. (2) CE. (3) Pediatric patients. (4) Deceased patients who had not provided GC for reuse of their data.

### 2.3. Data Collection

Data of interest were systematically collected from patient medical records and anonymized before analysis.

Epidemiological data included patient demographics (sex and age), year of diagnosis, and place of residence at the time of diagnosis. For patients residing permanently in Switzerland, the canton of residence was recorded; for patients living abroad, the country of origin was documented.

Clinical data included presenting symptoms, underlying liver disease, and any history of Immunosuppression (IS). Patients were classified as immunosuppressed if there was documented evidence of immunosuppressive conditions or treatments prior to or at the time of AE diagnosis. IS was defined as any condition or therapy known to impair immune function, including hematologic or solid organ malignancies, human immunodeficiency virus (HIV) infection with CD4 count below 500 cells/mm^3^, and the use of immunosuppressive agents such as chemotherapy, calcineurin inhibitors, mycophenolate mofetil, methotrexate, monoclonal antibodies, or prolonged corticosteroid therapy. Radiological data included imaging modalities used for diagnosis (US, CT, MRI, PET-CT), characteristics of liver lesions (type and size of the largest lesion), presence of metastases, and metabolic activity on PET-CT when available.

Laboratory parameters at diagnosis included liver function tests (AST, ALT, GGT and ALP), and liver function (albumin, total bilirubin and PT), inflammatory markers (CRP and eosinophil count), renal function (total creatinine) and echinococcosis serology (EgHF, Em2, Em18 via ELISA and species-specific immunoblot assays for AE and CE).

When available, histopathological data were reviewed to confirm the diagnosis of AE in tissue samples.

Treatment-related data included surgical intervention, medical therapy with benzimidazoles (albendazole or mebendazole), and a treatment abstention strategy (“watch and wait”) in palliative settings.

Last follow-up included the year of the last clinical assessment and categorized outcomes as remission, stable disease, or progression. Remission was defined as either the complete disappearance of all hepatic lesions on morphological imaging or a significant reduction in total lesion volume, accompanied by negative Em18 serology and metabolic inactivity on PET-CT. Stable disease was defined as a change in total lesion volume of less than approximately 20%, with persistently positive serology and no evidence of new lesions. Progression was defined as an increase in total lesion volume or the detection of new hepatic or extrahepatic lesions on follow-up imaging. When follow up data was not available, outcome information was gathered by calling patients and or their general practitioner.

### 2.4. Statistical Analysis

Statistical analysis was carried out with the support of a dedicated biostatistician.

Descriptive statistics included mean and standard deviation (SD) or median and range for continuous variables as well as frequencies or percentages for categorical variables. The rate of AE was calculated as the number of cases divided by the total cantonal population for the same year. Its trend over time was analyzed using a robust linear regression model. The calculated beta coefficient and associated *p*-value gave an average estimate of a linear progression of cases and its statistical significance. A similar analysis was performed to quantify the shift in the median age at diagnosis over time. The immunosuppressed and immunocompetent patients were compared using Fisher’s exact test for binary variables and Mann–Whitney test for continuous variables. The strength of association between the immunosuppression and covariates was assessed using the odds radio (OR) and the calculated *p*-value. Analysis was performed using Stata software (StataCorp. 2023. Stata Statistical Software: Release 18. College Station, TX, USA: StataCorp LLC).

## 3. Results

### 3.1. Baseline Cohort Characteristics

**Figure 3:**
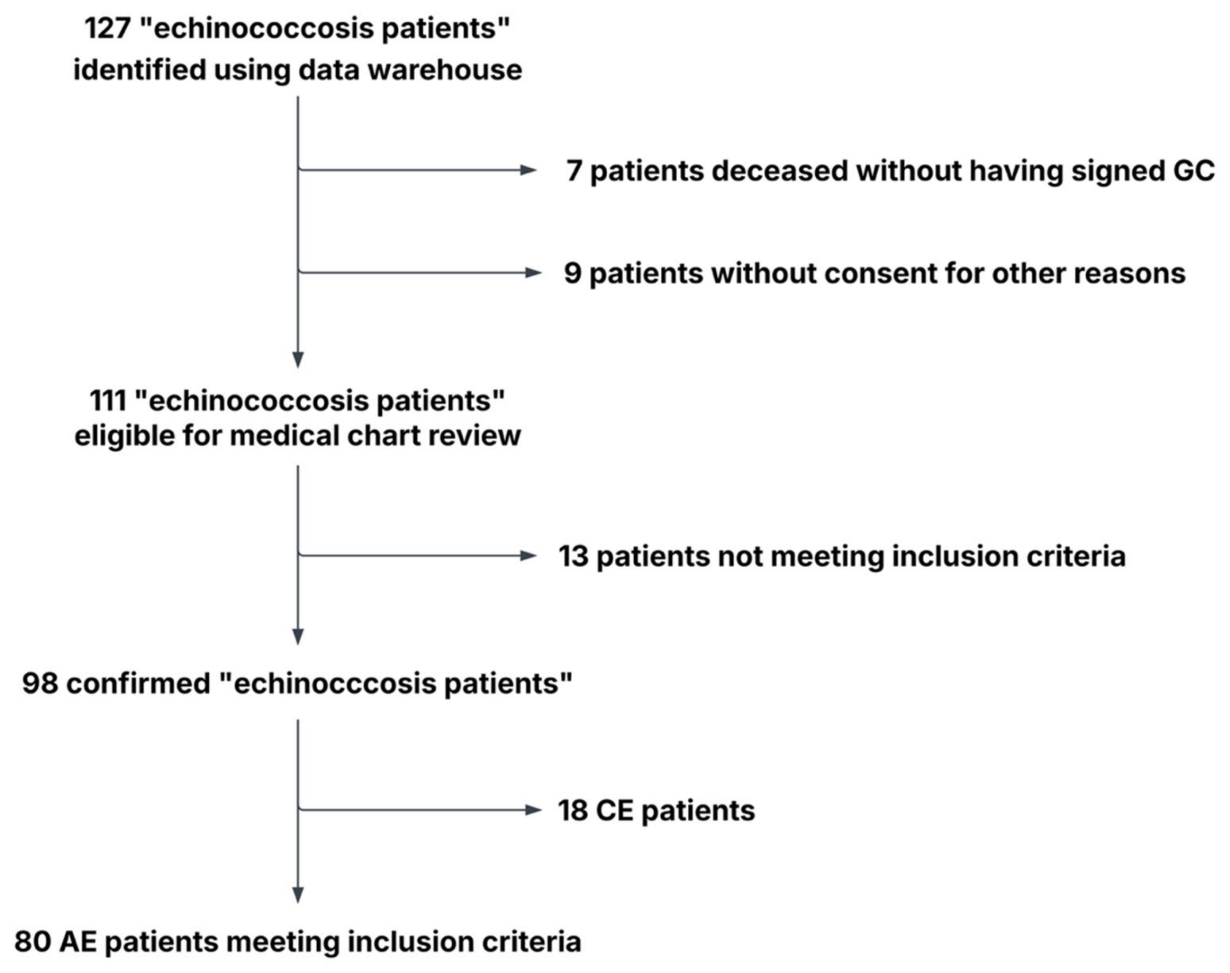
Flowchart portraying the patient selection process. Of 127 identified echinococcosis patients, 16 were excluded (7 deceased without consent, 9 due to lack of consent or other barriers). Of the remaining 111, 13 were excluded after chart review for not meeting inclusion criteria (diagnosis before 2010, unconfirmed diagnosis, or unclear type of echinococcosis). Among the 98 confirmed cases, 18 with CE were excluded, leaving a final cohort of 80 patients with AE.

A total of 80 eligible patients were identified during the study period from 1 January 2010 to 31 December 2023, with a median age at diagnosis of 61.5 years (range, 21–85) and an equal sex distribution (50.0% male). Figure 3 provides a summary of the patient selection process.

21.3% (17/80) of patients were immunosuppressed. The median size of the largest hepatic lesion was 6.0 cm (range, 1.0–18.0 cm). Multifocal liver involvement was observed in 55.1% (43/78) of cases, and extrahepatic disease in 20.5% (16/78). Histopathological confirmation was achieved in 86.3% (69/80) of patients, based on surgical specimens in 60.0% (48/80), combined surgical and biopsy material in 21.3% (17/80), and liver biopsy alone in 3.8% (3/80). Finally, one histological confirmation originated from a post-mortem liver sample, obtained during autopsy. The most common treatment modality was surgery combined with benzimidazole therapy (80.0%; 64/80), followed by benzimidazole monotherapy (16.3%; 13/80), “watch and wait” strategy (2.5%; 2/80) and surgery alone (1.3%; 1/80). One patient (1.3%) underwent liver transplantation (see Table 1).

**Figure 4:**
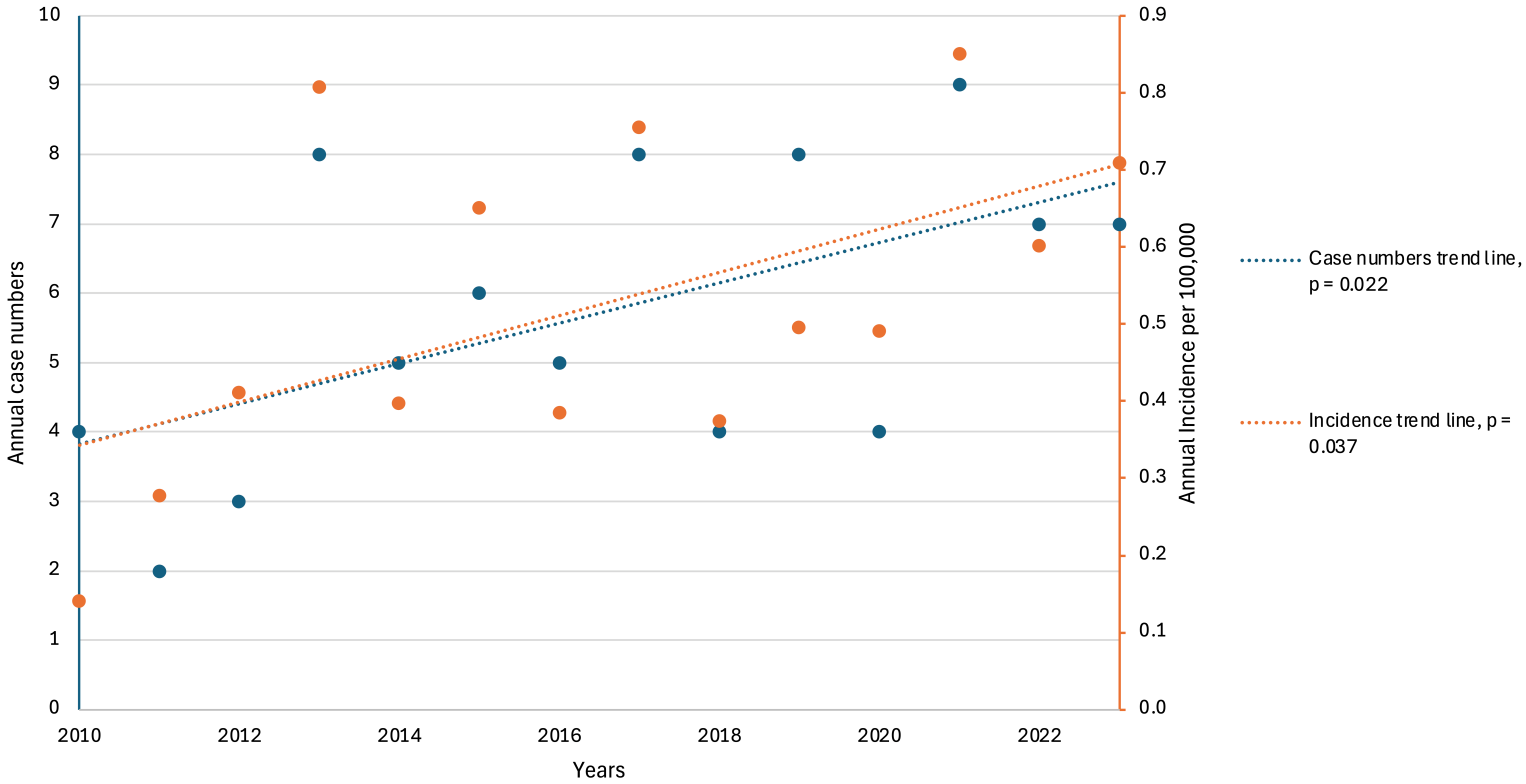
Annual case counts and incidence of AE (2010–2023). Annual cases are represented by blue dots, with robust linear regression (blue line) highlighting a statistically significant increase over the 13-year study period (coeff = 0.299; *p* = 0.022). Annual AE incidence in canton de Vaud is represented by orange dots, with robust linear regression (orange line) also indicating a statistically significant increase (coeff = 0.032; *p* = 0.037).

Annual case counts were analyzed using robust linear regression to assess temporal trends across the full cohort (N = 80), revealing a statistically significant increase in the number of patients diagnosed and treated annually at CHUV between 2010 and 2023 (coeff = 0.299; *p* = 0.022; Figure 4).

Of the 80 patients, 58 (72.5%) were residents of the Canton of Vaud, Switzerland, while the remainder were referred from neighboring regions (Canton de Fribourg, Canton de Neuchâtel and Jura). Based on official cantonal population data from 2010 to 2023, the mean annual incidence was 0.53 cases per 100,000 population, with annual rates ranging from 0.14 to 0.85 per 100,000. A statistically significant increase in the incidence of new cases over time was observed (coeff = 0.032; *p* = 0.037).

**Figure 5:**
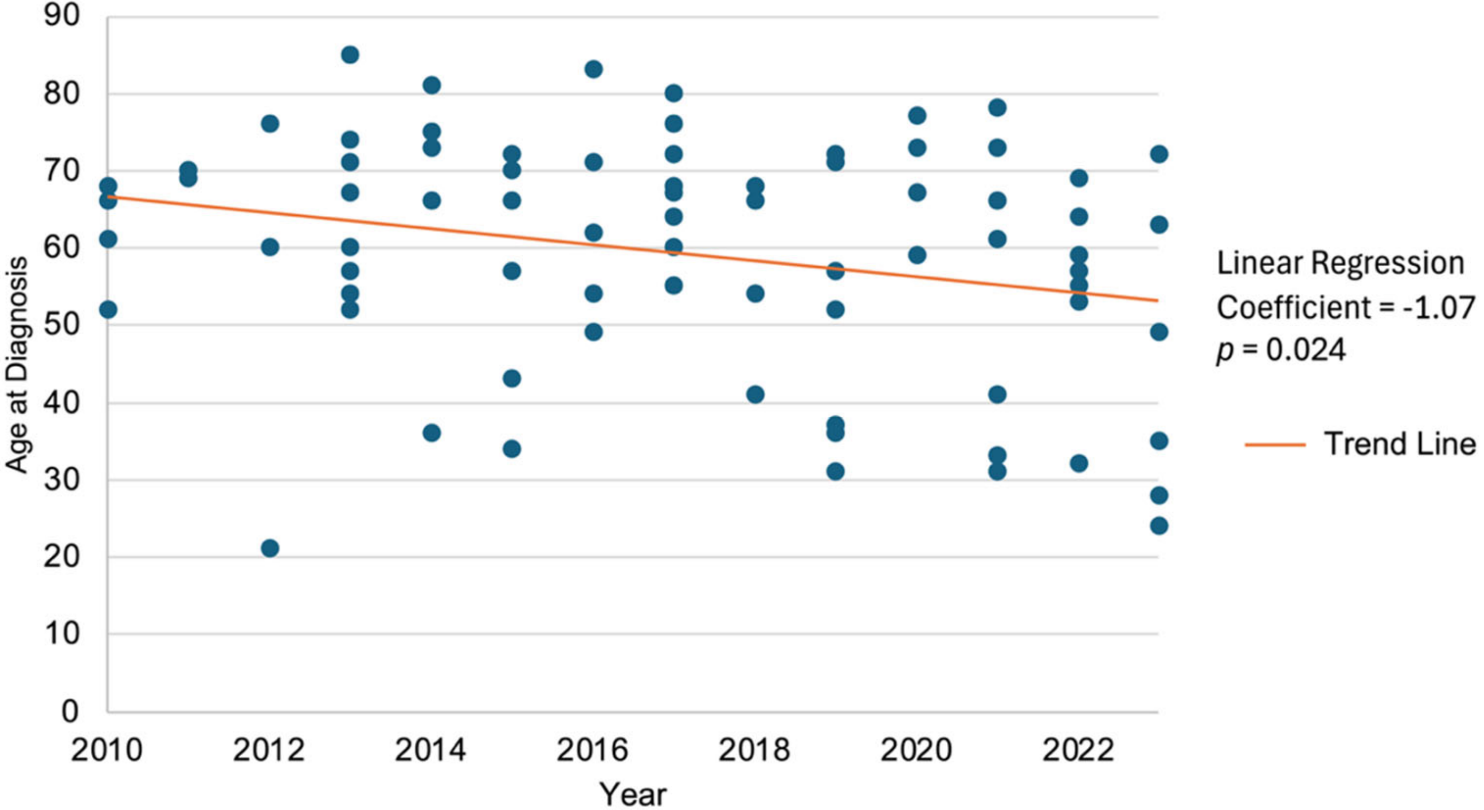
Evolution of age at diagnosis of AE cases (2010–2023). Age at diagnosis is represented by blue dots, with robust linear regression (orange line) showing a statistically significant decrease over the 13-year study period, corresponding to an estimated decline of approximately 1 year per year (coeff = −1.07; *p* = 0.024).

The median age at diagnosis declined significantly between 2010 and 2023, as shown by robust regression analysis (coeff = −1.07; *p* = 0.024, Figure 5).

### 3.2. Clinical Presentation

At the time of diagnosis, 35 patients (43.8%) were symptomatic, while 45 (56.2%) were asymptomatic. Jaundice and/or cholangitis were the initial symptoms presenting in 9 patients (11.3%).

Seventeen patients (21.3%) were immunosuppressed at the time or prior to AE diagnosis. Of these, 8 (47.1%) had a history of oncologic disease treated with chemotherapy, another 8 (47.1%) were receiving immunosuppressive therapy for conditions such as autoimmune diseases or solid organ transplantation, and 1 patient (5.9%) was HIV-positive with a history of CD4 count below 500 cells/mm^3^. The proportion of immunosuppressed patients remained stable over time, with 7 cases in 2010–2015, 5 in 2016–2019, and 5 in 2020–2023. Another 4 patients (5.0%) had a documented history of cirrhosis (see Table 2).

### 3.3. Radiology

Radiological examinations were available for review in 78 patients. At diagnosis, 35 patients (44.9%) had a single hepatic lesion, while 43 (55.1%) had multifocal liver disease. Extrahepatic disease was observed in 16 patients (20.0%) of whom 12 (15.0%) had extension to adjacent organs and/or regional lymph nodes, while 4 (5.1%) had distant “metastases”. These included one patient with pancreatic and pulmonary lesions, one with a splenic lesion, one with diffuse peritoneal involvement, and one with extensive inferior vena cava invasion extending into the right heart. The median size of the largest hepatic lesion across the cohort was 6.0 cm (range: 1.0–18.0).

**Figure 6:**
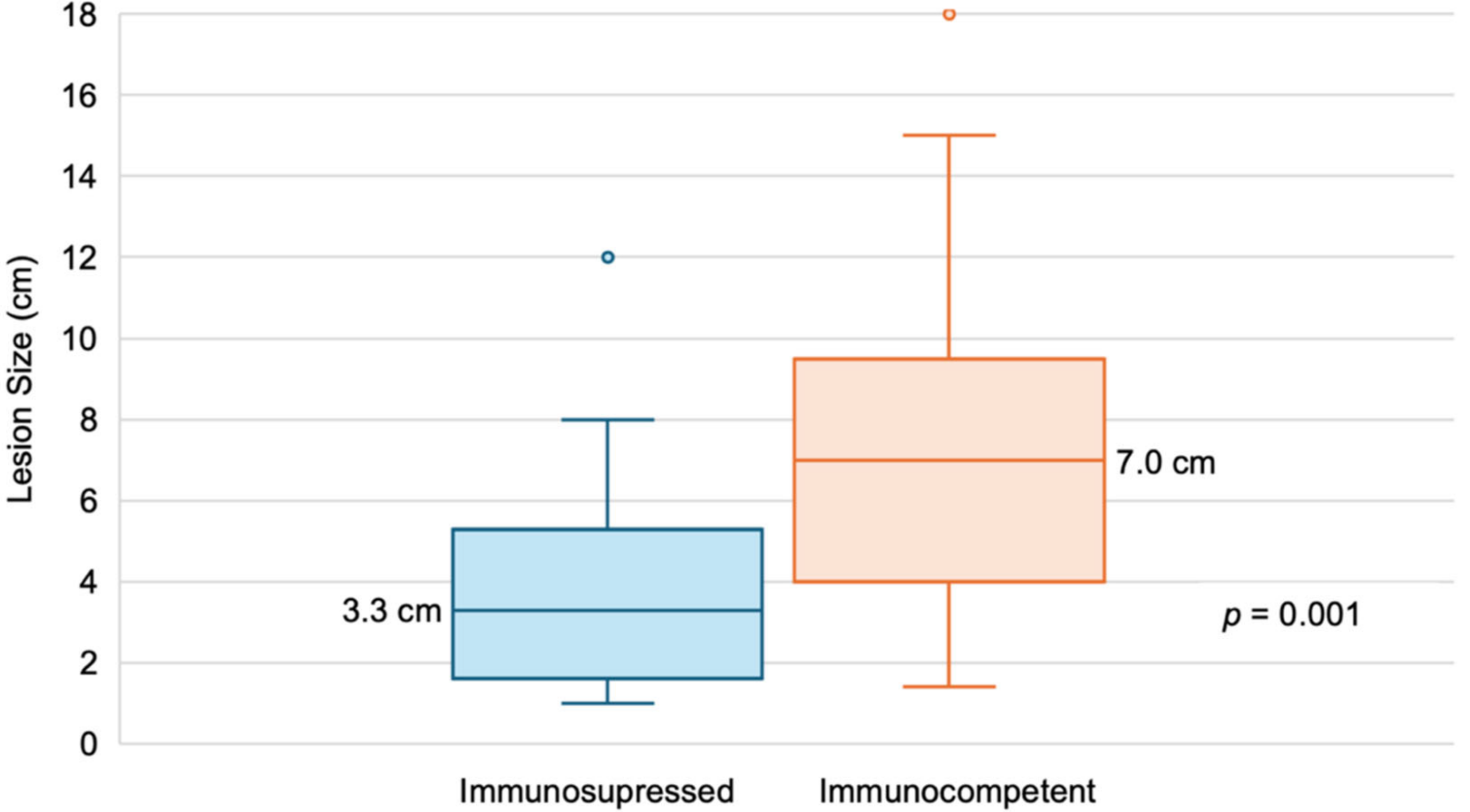
Comparison of lesion size by immunosuppression status. Box plots showing the largest lesion size at diagnosis in immunosuppressed (n = 17) and immunocompetent (n = 61) patients. Immunosuppressed individuals had significantly smaller lesions (*p* = 0.001). Among the immunosuppressed patients, the median lesion size was 3.3 cm (range: 1.0–12.0) as compared to 7.0 cm (range: 1.4–18.0) in immunocompetent patients.

The median size of the largest lesion differed significantly between immunosuppressed and immunocompetent patients respectively (*p* = 0.001; Figure 6). Additionally, 41.2% (7/17) of immunosuppressed patients presented with multiple hepatic lesions, compared to 59.0% (36/61) of immunocompetent individuals.

### 3.4. Serology

Serological data were available for 75 out of 80 patients. Out of these, 55 (73.3%) had serology supporting AE (positive Em2, Em18, and/or EM specific immunodot), 17 (22.7%) had non-specific echinococcosis serology, and 3 patients (4%) had negative echinococcosis serology, despite a confirmed AE diagnosis based on imaging and histology. Two out of the three patients (66.6%) with negative echinococcosis serology were immunosuppressed.

### 3.5. Histopathology

Biopsy was significantly more frequent in patients without AE-specific serology, including those with non-specific echinococcosis serology, negative serology, or unavailable serological data. In this group, the biopsy rate was 44.0% (11/25), compared to 16.4% (9/55) in patients with EM specific serology (*p* = 0.012; OR = 4.02).

### 3.6. Therapy

Among the 65 patients who underwent surgery at the CHUV visceral surgery department, 40 (61.5%) achieved complete (R0) resection. Of the 15 patients who did not undergo surgery, 6 (40.0%) were deemed inoperable due to advanced disease. In the remaining 9 cases (60.0%), surgery was withheld for various reasons, including patient refusal, advanced age, favorable response to medical therapy prior to scheduled intervention, and, in one case, severe malnutrition.

One liver transplantation was performed during the study period in a patient with advanced liver infiltration and intolerance to benzimidazoles.

### 3.7. Outcome

Follow-up data were available for 74 of the 80 patients (92.5%), with a median duration from diagnosis to last follow-up of 3 years (range, 0–12 years). Among the 74 patients with available follow-up, 50 (67.6%) achieved remission, 19 (25.7%) had stable disease, and 5 (6.8%) demonstrated disease progression. Four of the five patients with progressive disease had discontinued benzimidazole therapy due to intolerance, including one patient who experienced relapse following an incomplete (R1) surgical resection. The remaining case of disease progression occurred in the context of poor treatment adherence.

## 4. Discussion

With this study, we aimed to characterize AE cases managed at our tertiary care center (CHUV), where each case is systematically reviewed by a dedicated multidisciplinary team, including hepatobiliary surgeons, radiologists, interventional radiologists, infectiologists, and hepatologists. During these multidisciplinary board discussions, clinical observations raised concerns about a rising incidence of AE and an increasing case of severe AE in younger patients, underscoring the need for systematic characterization.

In our study, the mean annual incidence of AE in the Canton of Vaud was 0.53 per 100,000 population between 2010 and 2023. This represents a clear increase compared to earlier national estimates [13]. More recently, the Swiss Echinococcosis Network reported an average of 51 new cases per year in 2020–2021, corresponding to an estimated incidence of 0.58 to 1.33 per 100,000 population when including serological cases [25]. These more recent figures align with our findings and confirm that Switzerland is among the European countries with the highest incidence of AE [5].

Several factors may explain the increasing incidence of AE observed in Switzerland. A key factor appears to be the marked rise in fox populations since the 1980s, which nearly quadrupled by the mid-1990s [14]. This growth, coupled with high EM infection rates among foxes has played a central role [10,13]. Moreover, the urbanization of foxes and their spread into peri-urban and suburban environments have brought these wildlife reservoirs into closer contact with human populations, thereby increasing the risk of human transmission [10,26]. Considering the long latency period of AE, these ecological changes have most likely contributed to the emergence of a growing AE epidemic since the early 21st century.

Our median age at diagnosis (61.5 years) was slightly higher than reported in previous European studies, including Switzerland [15,27,28]. Interestingly, we observed a statistically significant decrease in patient age at diagnosis, with an estimated decrease of approximately 1 year per year over the studied period.

Our clinical findings provide additional insights into the evolving presentation of AE in Switzerland. In our cohort, 56% of patients were asymptomatic at the time of diagnosis, a proportion notably higher than in earlier studies from France and Germany [5]. Moreover, A cohort study from Zurich showed a marked temporal increase in asymptomatic presentation, increasing from 20% in 1973–2002 to 54% in 2013–2022 [27]. Our results corroborate this temporal shift and are likely explained by the growing use and improved sensitivity of cross-sectional imaging techniques, which have substantially contributed to an increase in cases of incidental AE diagnosis [29]. This trend, alongside ecological factors, may also contribute to the rising incidence of AE in recent years.

Our results also highlight the possible diagnostic challenges posed by AE, particularly in cases where serological results are non-specific or negative. As expected, biopsy was performed significantly more often in patients without EM specific serology (44.0%) as compared to those with EM specific serology (16.4%). This indicates that biopsy remains a relevant diagnostic tool in particular cases, where serology is not contributive which seems to be more frequent in early disease stages [30]. These results highlight the importance of a combined diagnostic approach, integrating imaging, serology, and histology to ensure accurate diagnosis of AE.

In our cohort, 65 of 80 patients (81.3%) underwent surgical intervention, a higher proportion than reported in major European AE studies, where intervention rates ranged from 43% to 57% and have declined in recent years [15,27,31]. First, we considered whether the high surgical rate in our cohort might reflect earlier disease detection, as suggested by the high proportion of asymptomatic patients. However, this explanation appears insufficient, as prior studies show that even with earlier detection, surgery rates tend to decline [27,31]. An alternative explanation for the high surgical intervention rate could lie in local treatment practices: the visceral surgery service at CHUV has a long-standing expertise in hepatic surgery for AE, often favoring this definitive treatment approach over lifelong benzimidazole therapy. This institutional preference may have contributed to a broader surgical indication spectrum, potentially explaining the high rate of surgical intervention in our cohort.

Since the beginning of the 21st century, a major increase in the number of AE patients with associated immunosuppressive conditions has been observed. Several studies have highlighted this trend, with the proportion of immunosuppressed AE patients rising from well below 5% before 2000 to approximately 20% in more recent decades [17,32]. Immunosuppressed patients are not only at elevated risk for developing AE [16], but also tend to experience faster disease progression compared to immunocompetent AE patients [32,33].

Given these concerns, we were particularly interested in examining this population.

In our cohort, immunosuppressed patients accounted for 21.3% (17/80) of total cases. However, we did not observe an increase in the proportion of immunosuppressed patients over the study period, unlike in earlier series. This finding is consistent with a recent Zurich study (2000–2021), which similarly reported increasing AE case numbers without a corresponding rise in immunosuppressed AE cases [34]. These observations suggest that the current increase in AE incidence is unlikely to be driven by immunosuppression-associated conditions.

In the immunosuppressed subgroup, only 17.6% of patients were symptomatic at diagnosis, a proportion significantly lower than in immunocompetent patients. Additionally, the median size of the largest lesion was significantly smaller in immunosuppressed patients, suggesting a lower disease burden at diagnosis in this population. Similar findings have been reported in other publications [16,32,34]. This pattern is likely due to earlier detection, as immunosuppressed patients undergo more frequent medical evaluations and imaging, leading to incidental AE findings at earlier stages.

Another noteworthy observation in this group was the proportionally high prevalence of negative serologies (14.3%, 2/14) when compared to the immunocompetent group (1.6%, 1/61), which was also reported in previous studies [16,32]. It is important to consider that negative serologies in immunosuppressed patients may reflect not only the effect of their immunosuppression but also smaller lesion size and earlier disease stage, both of which are known to decrease serology’s sensitivity [30,35].

The evolving epidemiology observed in this study underscores the need for greater awareness of AE. To reduce diagnostic delays, clinicians should maintain a high index of suspicion when encountering incidental, atypical, or unexplained hepatic lesions, even in younger patients. Given the rarity and complexity of AE, along with the specialized requirements for long-term management, early referral to expert centers is recommended to ensure standardized care and optimal outcomes.

## 5. Conclusions

In summary, this study provides important insights into the changing epidemiology and clinical presentation of AE in Switzerland. We observed a clear increase in incidence over the past decade, including a potential increase among younger patients, accompanied by a high proportion of asymptomatic cases, likely reflecting earlier detection through improved and more frequent use of imaging. Among immunosuppressed AE patients, we identified a lower disease burden at diagnosis, yet the proportion of cases no longer appears to be increasing, in contrast to earlier trends. Together, these findings underscore the need for continued surveillance and prevention. However, the relatively low number of observations in this regional cohort limits the statistical power for more granular temporal analyses. To overcome these limitations and strengthen national epidemiological monitoring, the implementation of a national registry with mandatory AE case reporting should be strongly considered to guide future public health efforts.

## Figures and Tables

**Table 1:** Demographic and clinical characteristics of 80 AE patients.

Variable	Value
Median age at diagnosis in years (range)	61.5 (21–85)
Male, n (%)	40 (50.0)
Symptomatic at diagnosis, n (%)	35 (43.8)
Cholangitis and/or jaundice at presentation, n (%)	9 (11.3)
Immunosuppressed patients, n (%)	17 (21.3)
Liver cirrhosis, n (%)	4 (5.0)
Radiology ^1^, n (%)	78 (97.5)
Median size of largest lesion in cm (range)	6.0 (1.0–18)
Multifocal liver disease, n (%)	43 (55.1)
Extra-hepatic disease ^2^, n (%)	16 (20.5)
Histopathological confirmation, n (%)	69 (86.3)
Surgical specimen, n (%)	48 (60.0)
Surgical specimen and liver biopsy, n (%)	17 (21.3)
Liver biopsy alone, n (%)	3 (3.8)
Post-mortem specimen, n (%)	1 (1.4)
Treatments ^3^, n (%)	80 (100)
Surgery combined with benzimidazole, n (%)	64 (80.0)
Benzimidazole alone, n (%)	13 (16.3)
Surgery alone, n (%)	1 (1.3)
Watch and wait, n (%)	2 (2.5)
Liver transplantation, n (%)	1 (1.3)
Outcomes ^4^, n (%)	74 (92.5)
Remission, n (%)	50 (67.6)
Stable disease, n (%)	19 (25.7)
Progressive disease, n (%)	5 (6.8)

^1^ Radiological data were available for 78/80 patients. ^2^ Including invasion of adjacent organs, lymph nodes, or distant metastases. ^3^ All patients treated via benzimidazole received albendazole as first line treatment. It was replaced by mebendazole in cases of toxicity of intolerance. ^4^ Outcome data were available for 74/80 patients. Remaining 6 patients were lost to follow-up at our center.

**Table 2:** Comparison of clinical characteristics between immunosuppressed and immunocompetent patients. Statistically significant differences are highlighted in bold text.

Variable		Immunocompetent	Immunosuppressed	*p*-Value (OR ^1^)
Total number of patients, n (%)		63 (79)	17 (21)	
Demography	age in years, median (range)	64 (21–83)	60 (28–85)	0.37
Clinical presentation	Symptomatic disease, n (%) n = 38	32 (50.8)	3 (17.6)	**0.026** (0.21)
Radiology ^2^	Size of largest lesion in cm, median (range)	7 (1.4–18.0)	3.3 (1.0–12.-0)	**0.001**
	Multifocal liver disease, n (%) n = 43	36 (59.0)	7 (41.2)	0.27 (0.49)
	Extra-hepatic disease, n (%) n = 16	15 (24.6)	1 (5.9)	0.17 (0.20)
Serology ^3^	Negative, n (%)	1 (1.6)	2 (14.3)	0.12 (8.0)
Pathology	Biopsy, n (%) n = 20	13 (20.6)	7 (41.2)	0.11 (2.69)
Therapy ^4^	Surgery combined with benzimidazole, n (%) n = 64	51 (81.0)	13 (76.5)	0.74 (0.76)
	Benzimidazole alone, n (%) n = 14	10 (15.9)	4 (23.5)	0.48 (1.63)
Outcome ^5^	Remission, n (%) n = 50	39 (66.1)	11 (73.3)	0.34 (2.15)

^1^ OR were calculated using the immunocompetent group as the reference. ^2^ Radiological data was available for 78/80 patients. ^3^ Serological data at time of diagnosis were available for 75/80 patients. ^4^ The remaining 2 patients, treated with either a watch-and-wait approach or surgery alone, were both immunocompetent. ^5^ Outcome data were available for 74/80 patients. Remaining 6 patients were lost to follow-up, of whom 4 were immunocompetent and 2 immunosuppressed.

## Data Availability

Data cannot be shared publicly because of confidentiality reasons. Data are available through the Ethics Committee of canton de Vaud (CER-VD) for researchers who meet the criteria for access to confidential data.

## Abbreviations

AE	Alveolar Echinococcosis
ALP	Alkaline Phosphatase
ALT	Alanine Aminotransferase
AST	Aspartate Aminotransferase
CE	Cystic Echinococcosis
CRP	C-Reactive Protein
CT	Computed Tomography
EM	Echinococcus Multilocularis
GC	General Consent
GGT	Gamma-Glutamyl Transferase
HIV	Human Immunodeficiency Virus
MRI	Magnetic Resonance Imaging
OR	Odds Ratio
PET-CT	Positron Emission Tomography-Computed Tomography
PT	Prothrombin Time
US	Ultrasound
WHO	World Health Organization

## References

[1] Gottstein B., Stojkovic M., Vuitton D.A., Millon L., Marcinkute A., Deplazes P. (2015). Threat of alveolar echinococcosis to public health—A challenge for Europe. Trends Parasitol..

[2] World Health Organization (2024). Echinococcosis.

[3] Torgerson P.R., Devleesschauwer B., Praet N., Speybroeck N., Willingham A.L., Kasuga F., Rokni M.B., Zhou X.-N., Fèvre E.M., Sripa B. (2015). World Health Organization estimates of the global and regional disease burden of 11 foodborne parasitic diseases, 2010: A data synthesis. PLoS Med..

[4] Kern P., Bardonnet K., Renner E., Auer H., Pawlowski Z., Ammann R.W., Vuitton D.A., Kern P. (2003). European echinococcosis registry: Human alveolar echinococcosis, Europe, 1982–2000. Emerg. Infect. Dis..

[5] Lundström-Stadelmann B., Rostami A., Frey C.F., Torgerson P.R., Riahi S.M., Bagheri K., Kaethner M., Lachenmayer A., Beldi G., Gasser R.B. (2025). Human alveolar echinococcosis-global, regional, and national annual incidence and prevalence rates. Clin. Microbiol. Infect..

[6] Deplazes P., Rinaldi L., Alvarez Rojas C.A., Torgerson P.R., Harandi M.F., Romig T., Antolova D., Schurer J.M., Lahmar S., Cringoli G. (2017). Global distribution of alveolar and cystic echinococcosis. Adv. Parasitol..

[7] Casulli A., Abela B., Petrone D., Šoba B., Dezsényi B., Karamon J., Millon L., Saarma U., Antolová D., Chappuis F. (2026). Unveiling the incidences and trends of alveolar echinococcosis in Europe: A systematic review from the KNOW-PATH project. Lancet Infect. Dis..

[8] Craig P.S., Hegglin D., Lightowlers M.W., Torgerson P.R., Wang Q. (2017). Echinococcosis: Control and prevention. Adv. Parasitol..

[9] Combes B., Comte S., Raton V., Raoul F., Boué F., Umhang G., Favier S., Dunoyer C., Woronoff N., Giraudoux P. (2012). Westward spread of Echinococcus multilocularis in foxes, France, 2005–2010. Emerg. Infect. Dis..

[10] Liccioli S., Giraudoux P., Deplazes P., Massolo A. (2015). Wilderness in the ‘city’ revisited: Different *urbes* shape transmission of *Echinococcus multilocularis* by altering predator and prey communities. Trends Parasitol..

[11] Hegglin D., Bontadina F., Deplazes P. (2015). Human–wildlife interactions and zoonotic transmission of *Echinococcus multilocularis*. Trends Parasitol..

[12] European Food Safety Authority (2015). E. multilocularis Infections in Animals.

[13] Schweiger A., Ammann R.W., Candinas D., Clavien P.-A., Eckert J., Gottstein B., Halkic N., Muellhaupt B., Prinz B.M., Reichen J. (2007). Human alveolar echinococcosis after fox population increase, Switzerland. Emerg. Infect. Dis..

[14] Gloor S., Bontadina F., Hegglin D., Deplazes P., Breitenmoser U. (2001). The rise of urban fox population in Switzerland. Mamm. Biol..

[15] Piarroux M., Piarroux R., Giorgi R., Knapp J., Bardonnet K., Sudre B., Watelet J., Dumortier J., Gérard A., Beytout J. (2011). Clinical features and evolution of alveolar echinococcosis in France from 1982 to 2007: Results of a survey in 387 patients. J. Hepatol..

[16] Autier B., Gottstein B., Millon L., Ramharter M., Gruener B., Bresson-Hadni S., Dion S., Robert-Gangneux F. (2023). Alveolar echinococcosis in immunocompromised hosts. Clin. Microbiol. Infect..

[17] Lachenmayer A., Gebbers D., Gottstein B., Candinas D., Beldi G. (2019). Elevated incidence of alveolar echinococcosis in immunocompromised patients. Food Waterborne Parasitol..

[18] Wen H., Vuitton L., Tuxun T., Li J., Vuitton D.A., Zhang W., McManus D.P. (2019). Echinococcosis: Advances in the 21st century. Clin. Microbiol. Rev..

[19] Brunetti E., Kern P., Vuitton D.A. (2010). Writing Panel for the WHO-IWGE. Expert consensus for the diagnosis and treatment of cystic and alveolar echinococcosis in humans. Acta Trop..

[20] Torgerson P.R., Schweiger A., Deplazes P., Pohar M., Reichen J., Ammann R.W., Tarr P.E., Halkik N., Müllhaupt B. (2008). Alveolar echinococcosis: From a deadly disease to a well-controlled infection. Relative survival and economic analysis in Switzerland over the last 35 years. J. Hepatol..

[21] Joliat G.-R., Roulin D., Labgaa I., Uldry E., Demartines N., Halkic N., Melloul E. (2023). Nouveautés dans la prise en charge de l’échinococcose alvéolaire. Rev. Médicale Suisse.

[22] Pavlidis E.T., Galanis I.N., Pavlidis T.E. (2025). Current considerations for the management of liver echinococcosis. World J. Gastroenterol..

[23] Ambregna S., Koch S., Sulz M.C., Grüner B., Öztürk S., Chevaux J.B., Sulima M., De Gottardi A., Napoléon B., Abergel A. (2017). A European survey of perendoscopic treatment of biliary complications in patients with alveolar echinococcosis. Expert Rev. Anti-Infect. Ther..

[24] Koch S., Bresson-Hadni S., Miguet J.-P., Crumbach J.-P., Gillet M., Mantion G.-A., Heyd B., Vuitton D.-A., Minello A., Kurtz S. (2003). Experience of liver transplantation for incurable alveolar echinococcosis: A 45-case European collaborative report. Transplantation.

[25] Universimed Alveolar Echinococcosis in Switzerland. https://www.universimed.com/ch/article/gastroenterologie/alveolar-switzerland-265336.

[26] Deplazes P., Hegglin D., Gloor S., Romig T. (2004). Wilderness in the city: The urbanization of *Echinococcus multilocularis*. Trends Parasitol..

[27] Deibel A., Kindler Y., Mita R., Ghafoor S., Meyer Zu Schwabedissen C., Brunner-Geissmann B., Schweiger A., Grimm F., Reinehr M., Weber A. (2025). Comprehensive survival analysis of alveolar echinococcosis patients, University Hospital Zurich, Zurich, Switzerland, 1973–2022. Emerg. Infect. Dis..

[28] Ollagnon M., Bresson-Hadni S., Spahr L., Rubbia-Brandt L., Toso C., Chappuis F. (2025). Alveolar echinococcosis in the canton of Geneva between 2010 and 2021: A descriptive analysis. Swiss Med. Wkly..

[29] Bresson-Hadni S., Delabrousse E., Blagosklonov O., Bartholomot B., Koch S., Miguet J.-P., André Mantion G., Angèle Vuitton D. (2006). Imaging aspects and non-surgical interventional treatment in human alveolar echinococcosis. Parasitol. Int..

[30] Bebezov B., Mamashev N., Umetaliev T., Ziadinov I., Craig P.S., Joekel D.E., Deplazes P., Grimm F., Torgerson P.R. (2018). Intense focus of alveolar echinococcosis, South Kyrgyzstan. Emerg. Infect. Dis..

[31] Grüner B., Kern P., Mayer B., Gräter T., Hillenbrand A., Barth T.E.F., Muche R., Henne-Bruns D., Kratzer W., Kern P. (2017). Comprehensive diagnosis and treatment of alveolar echinococcosis: A single-center, long-term observational study of 312 patients in Germany. GMS Infect. Dis..

[32] Chauchet A., Grenouillet F., Knapp J., Richou C., Delabrousse E., Dentan C., Millon L., Di Martino V., Contreras R., Deconinck E. (2014). Increased incidence and characteristics of alveolar echinococcosis in patients with immunosuppression-associated conditions. Clin. Infect. Dis..

[33] Vuitton D.A., Gottstein B. (2010). Echinococcus multilocularis and its intermediate host: A model of parasite-host interplay. BioMed Res. Int..

[34] Deibel A., Meyer zu Schwabedissen C., Husmann L., Grimm F., Deplazes P., Reiner C.S., Müllhaupt B. (2022). Characteristics and clinical course of alveolar echinococcosis in patients with immunosuppression-associated conditions: A retrospective cohort study. Pathogens.

[35] Hotz J.F., Peters L., Kapp-Schwörer S., Theis F., Eberhardt N., Essig A., Grüner B., Hagemann J.B. (2022). Evaluation of serological markers in alveolar echinococcosis emphasizing the correlation of PET-CTI tracer uptake with RecEm18 and echinococcus-specific IgG. Pathogens.

